# Supplementary material for: The Space Omics and Medical Atlas (SOMA) and international astronaut biobank
Source: Nature. 2024 Jun 11;632(8027):1145–54. doi: 10.1038/s41586-024-07639-y (PMC11357981; doi:10.1038/s41586-024-07639-y)
Supplement: Supplementary file 1 — This file contains Supplementary Figures 1–3, Supplementary Tables 4 and 9, Supplementary Note 1 and additional references. [file 41586_2024_7639_MOESM1_ESM.pdf]

nature portfolio

https://doi.org/10.1038/s41586-024-07639-y

Supplementary information

The Space Omics and Medical Atlas (SOMA) and international astronaut biobank

In the format provided by the authors and unedited

Supplementary Information for

The Space Omics and Medical Atlas (SOMA): A comprehensive data resource and biobank for astronauts

Eliah G. Overbey[1,2,3,4*], JangKeun Kim[1,2], Braden T. Tierney[1,2], Jiwoon Park[1,2], Nadia Houerbi[1,2], Alexander G. Lucaci[1,2], Sebastian Garcia Medina[1,2], Namita Damle[1], Deena Najjar[5], Kirill Grigorev[1,2], Evan E. Afshin[1,2], Krista A. Ryon[1], Karolina Sienkiewicz[2,6], Laura Patras[7,8], Remi Klotz[9], Veronica Ortiz[9], Matthew MacKay[6], Annalise Schweickart[2,6], Christopher R. Chin[1], Maria A. Sierra[6], Matias F. Valenzuela[10], Ezequiel Dantas[11,12], Theodore M. Nelson[13], Egle Cekanaviciute[14], Gabriel Deards[6], Jonathan Foox[1,2], S. Anand Narayanan[15], Caleb M. Schmidt[16,17,18], Michael A. Schmidt[16,17], Julian C. Schmidt[16,17], Sean Mullane[19], Seth Stravers Tigchelaar[19], Steven Levitte[19,20], Craig Westover[1], Chandrima Bhattacharya[6], Serena Lucotti[7], Jeremy Wain Hirschberg[1], Jacqueline Proszynski[1], Marissa Burke[1], Ashley Kleinman[1], Daniel J. Butler[1], Conor Loy[21], Omary Mzava[21], Joan Lenz[21], Doru Paul[22], Christopher Mozsary[1], Lauren M. Sanders[14], Lynn E. Taylor[23], Chintan O. Patel[24], Sharib A. Khan[24], Mir Suhail[24], Syed G. Byhaqui[24], Burhan Aslam[24], Aaron S Gajadhar[25], Lucy Williamson[25], Purvi Tandel[25], Qiu Yang[25], Jessica Chu[25], Ryan W. Benz[25], Asim Siddiqui[25], Daniel Hornburg[25], Kelly Blease[26], Juan Moreno[26], Andrew Boddicker[26], Junhua Zhao[26], Bryan Lajoie[26], Ryan T. Scott[27], Rachel R. Gilbert[27], San-huei Lai Polo[27], Andrew Altomare[26], Semyon Kruglyak[26], Shawn Levy[26], Ishara Ariyapala[28], Joanne Beer[28], Bingqing Zhang[28], Briana M. Hudson[29], Aric Rininger[29], Sarah E. Church[29], Afshin Beheshti[30,31], George M. Church[32], Scott M. Smith[33], Brian E. Crucian[33], Sara R. Zwart[34], Irina Matei[7,12], David C. Lyden[7,12], Francine Garrett-Bakelman[35,36], Jan Krumsiek[1,2,6], Qiuying Chen[37], Dawson Miller[37], Joe Shuga[38], Stephen Williams[38], Corey Nemec[38], Iwijn De Vlaminck[21], Steven Gross[37], Kelly L. Bolton[39], Susan M. Bailey[23,40], Richard Granstein[41], David Furman[10,42,43,44], Ari M. Melnick[12,22], Sylvain V. Costes[14], Bader Shirah[45], Min Yu[9], Anil S. Menon[34], Jaime Mateus[19], Cem Meydan[1,2,22*], Christopher E. Mason[1,2,3,46,47*]

Table of Contents

Supplementary Figures **4**
Supplementary Figure 1 4
Supplementary Figure 2 5
Supplementary Figure 3 7
Supplementary Tables **8**
Supplementary Table 1 8
Supplementary Table 2 8
Supplementary Table 3 8
Supplementary Table 4 8
Supplementary Table 5 10
Supplementary Table 6 10
Supplementary Table 7 10
Supplementary Table 8 10
Supplementary Table 9 10
Supplementary Note 1 .... **13**
I4 Data Compendium: Wet Lab Methodology **13**
Whole Genome Sequencing Extraction and Sequencing 13
Clonal Hematopoiesis Profiling 13
Biochemical and Blood Composition Measurements 13
Direct RNA-Seq Library Preparation and Sequencing 14
Single-Cell Gene Expression, Chromatin Accessibility, and Immune Profiling 14
cfRNA Extraction and Sequencing 15
Plasma LC/MS Untargeted Proteomics 15
Plasma-derived Extracellular Vesicle and Particle (EVP) Characterization 16
Plasma LC-MS/MS Untargeted Metabolomics 18
cfDNA Extraction and Sequencing 19
Telomere length assessment by multiplexed quantitative (q)PCR 20
Spatially Resolved Transcriptomics of Skin Tissue 21
Oral, Nasal, and Skin Swab Nucleic Acid Extraction 21
Metagenome and Metatranscriptome Sequencing 22
Cut&Run Sample Collection, Library Preparation, and Sequencing 22
I4 Data Compendium: Dry Lab Methodology **23**
WGS and cfDNA Data Processing 23
Clonal Hematopoiesis Data Processing 23
Single-Cell Gene Expression, Chromatin Accessibility, and Immune Profiling 24
Direct RNA-seq Data Processing 24
cfRNA Data Processing 24
Plasma Proteomic Data Processing 25
EVP Proteomic Data Processing 25
Metabolomics Data Processing 26

Spatially Resolved Transcriptomics of Skin Tissue Data Processing 27
Metagenome and Metatranscriptome Data Processing..27
Cut&Run Analysis 30

Supplementary Figures

Supplementary Figure 1

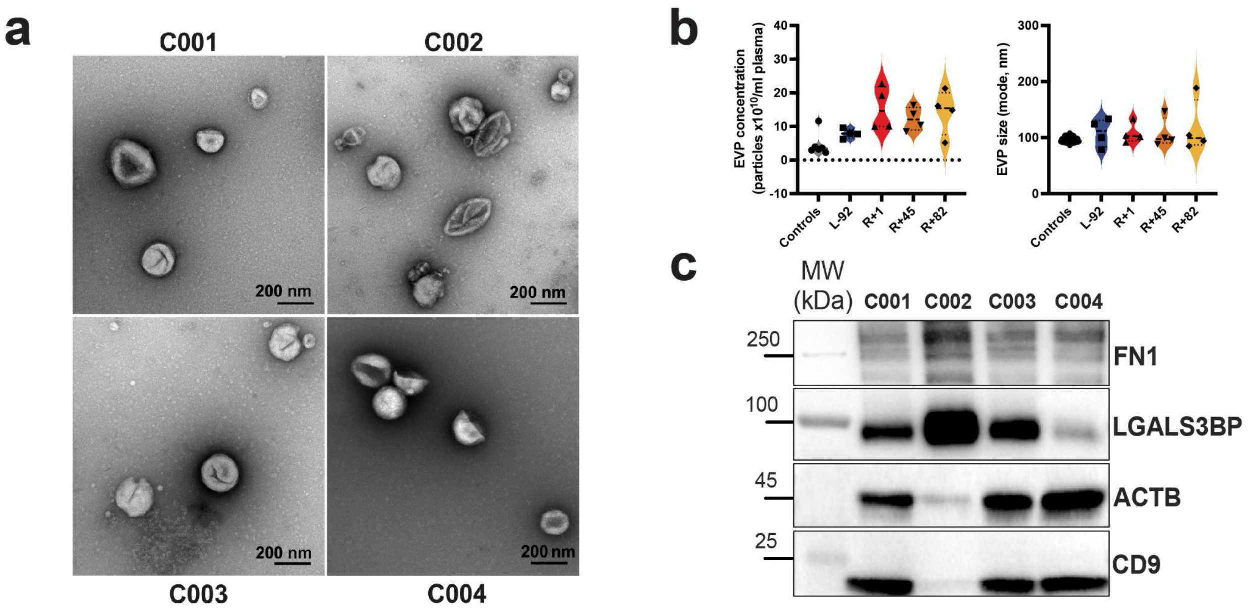

Supplementary Figure 1: Characterization of Plasma-circulating EVPs.
(a) Representative TEM images of EVPs from each of the crew members at the R+1 time point, scale bar = 200 nm. **(b)** NTA analysis for healthy controls from the NASA Twins Study (n=6)[24], pre-flight (L-92), and post-flight (R+1) and recovery (R+45, R+82) time points for all crew members showing plasma EVP concentration (particles x10^{10}/ml plasma) (left) and particle size represented as mode (nm) (right). **(c)** Pan-EVP marker Western blot of plasma-derived EVPs for C001 (L-92), C002 (L-3), C003 (L-44), and C004 (L-92). TEM and Westerns were performed once, due to limited starting material.

Supplementary Figure 2

a.

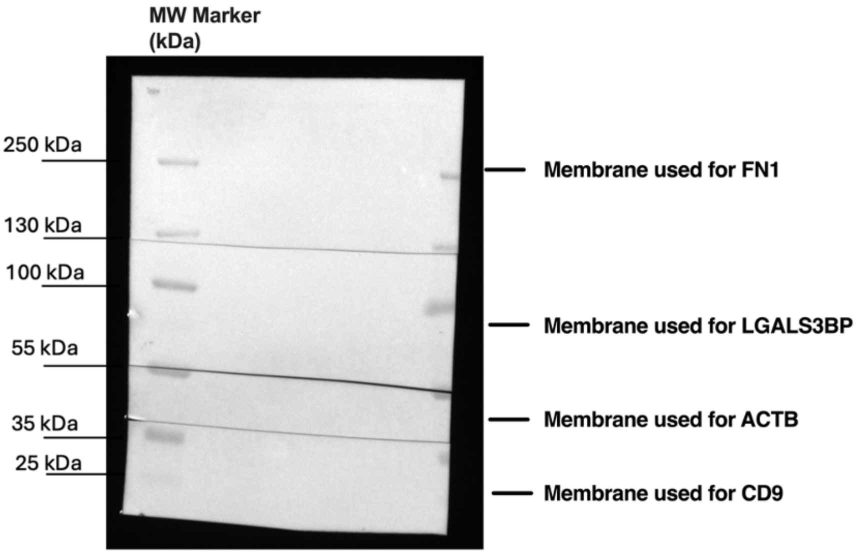

b.

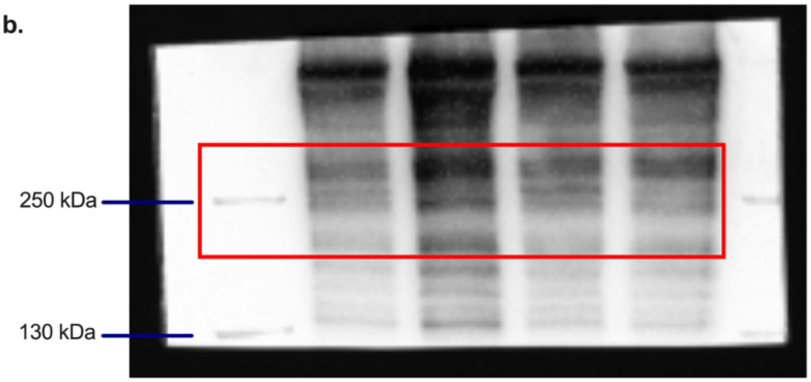

c.

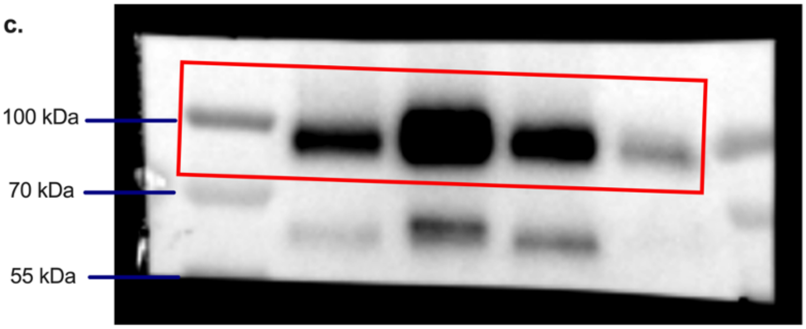

d.

e.

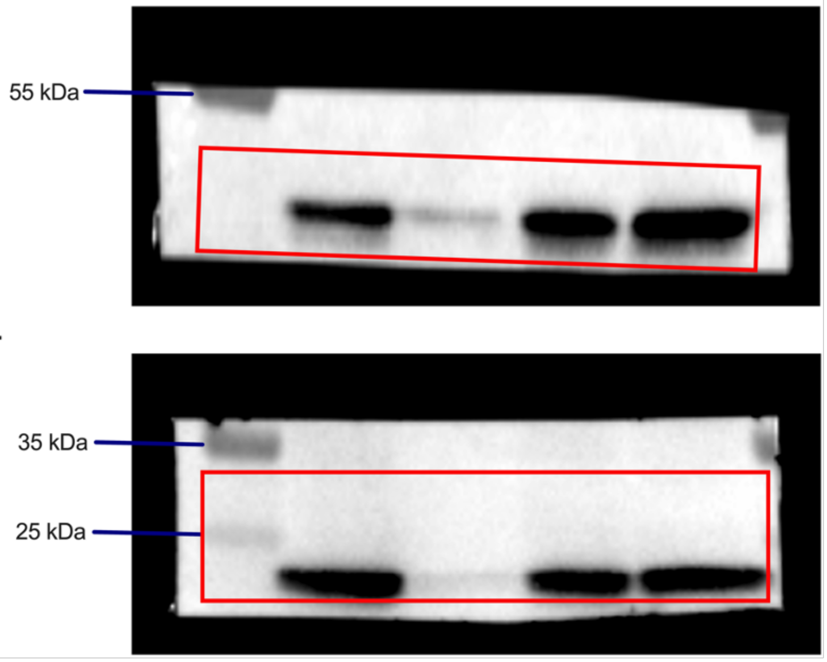

Supplementary Figure 2: Uncropped immunoblots for the western blot data.
Proteins were detected by western blotting, using the indicated antibodies and procedure in the Materials and methods section. The red rectangles indicate where the image was cropped for the final figure. All the EVP markers were detected from the same membrane: **a.** Image of the entire membrane indicating how it was cut for the detection of each target protein: FN1, LGALS3BP, ACTB, and CD9, respectively; **b.** Original image for FN1 protein, merged with the colorimetric image indicating the molecular weight marker; **c.** Original image for LGALS3BP, merged with the colorimetric image showing the molecular weight marker; **d.** Original image for ACTB, merged with the colorimetric image indicating the molecular weight marker; **e.** Original image for ACTB, merged with the colorimetric image showing the molecular weight markers.

Supplementary Figure 3

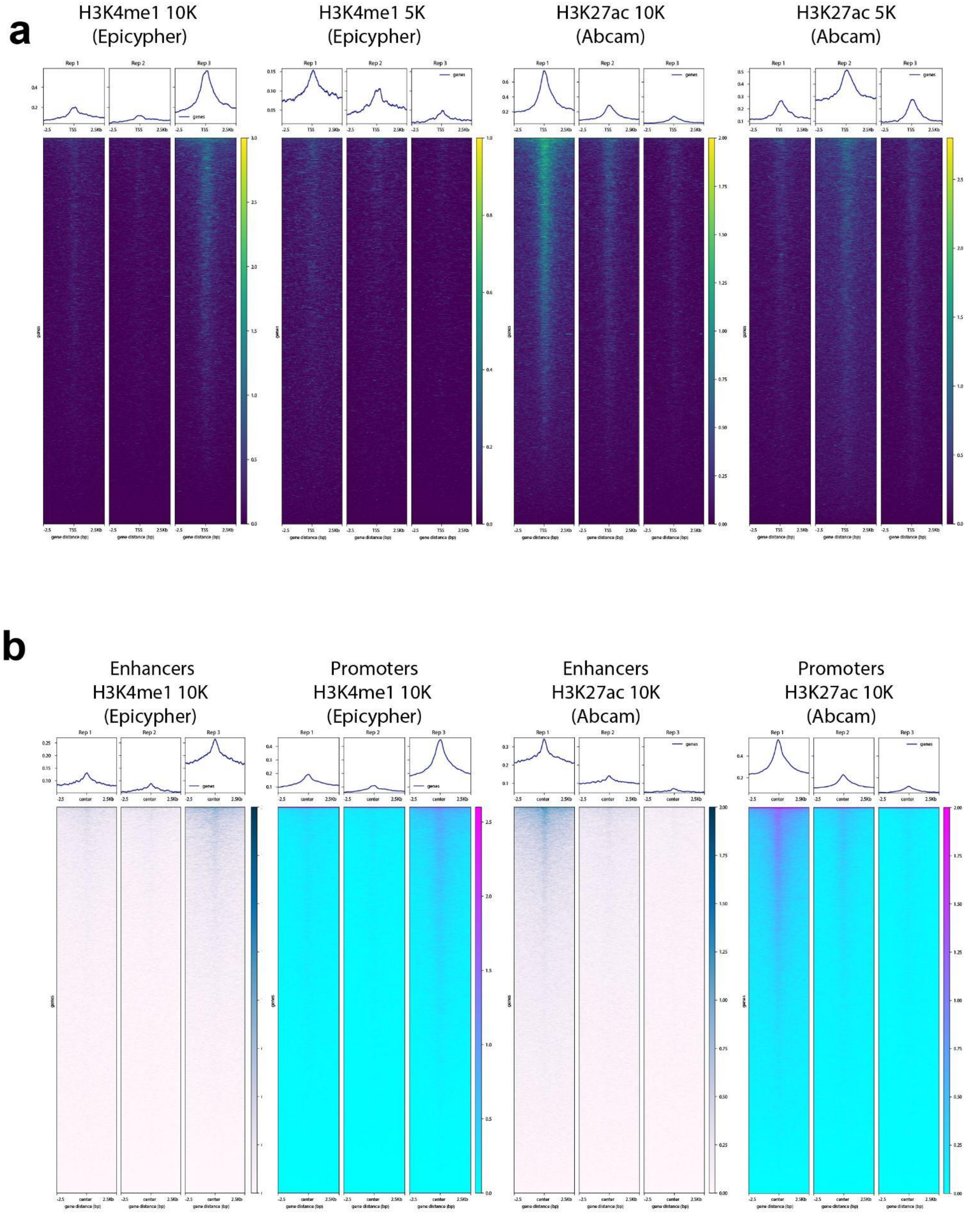

Supplementary Figure 3: Cut&Run Profiling for Histone Post-Translational Modifications. (a) Heatmaps were generated for histone PTMs H3K4me1 and H3K27ac for both 10K and 5K T-cells taken from C0002 crew member using above listed antibodies H3K4me1 Epicypher (13-0040) and Abcam H3K27ac (ab245911). Histone PTM signals were plotted across ±2kb of TSS of all genes belonging to GRCh38. **(b)** The same was done for hg38 Fantom Enhancer regions and hg38 Cage Promoter.

Supplementary Tables

Supplementary Table 1

Supplementary Table 1: Sample Information. Comprehensive list of samples collected from each crew member, at each timepoint, for each assay. Tab 1 is an overview of which samples are present at each timepoint. Tab 2 is an itemized list of each sample, including the number of sequenced DNA/RNA molecules for sequencing assays.

Supplementary Table 2

Supplementary Table 2: OSDR Studies. Comprehensive list of prior studies in OSDR for previous assays on human, metagenomic, and metatranscriptomic samples.

Supplementary Table 3

Supplementary Table 3: Sequencing and Mass Spectrometry Stats Tables. Sequencing and mass spectrometry statistics for multiome, TCR, BCR, cfRNA, dRNA, and proteomics assays.

Supplementary Table 4

Sample Type	Data Type	Profiles	Repository Link
Whole Blood	Direct RNA-seq	I4-FP1<br>I4-FP2<br>I4-FP3<br>I4-RP1<br>I4-RP2<br>I4-RP4<br>I4-RP5<br>I4-RP6<br>I4-LP1<br>I4-LP2<br>I4-LP4<br>I4-LP5<br>I4-LP6	https://osdr.nasa.gov/bio/repo/data/studies/OSD-569/
	RNA-seq	I4-FP1<br>I4-FP2<br>I4-FP3<br>I4-RP1<br>I4-RP2<br>I4-RP4<br>I4-RP5	

		I4-RP6<br>I4-LP1<br>I4-LP2<br>I4-LP4<br>I4-LP5<br>I4-LP6	
PBMC	Single-Nuclei RNA-seq	I4-FP1<br>I4-FP2<br>I4-RP1<br>I4-RP2<br>I4-RP3<br>I4-LP2<br>I4-LP3	https://osdr.nasa.gov/bio/repo/data/studies/OSD-570/
	Single-Nuclei ATAC-seq		
Plasma	Proteomics	I4-FP1<br>I4-FP2	https://osdr.nasa.gov/bio/repo/data/studies/OSD-571/
	Metabolomics	I4-FP1<br>I4-FP2<br>I4-RP3<br>I4-LP3	
	cfRNA	I4-FP1<br>I4-FP2<br>I4-FP3<br>I4-FP4<br>I4-RP1<br>I4-RP2<br>I4-RP3<br>I4-RP4<br>I4-RP5<br>I4-RP6<br>I4-LP1<br>I4-LP2<br>I4-LP3<br>I4-LP4<br>I4-LP5<br>I4-LP6	
EVPs	Proteomics	I4-FP1<br>I4-FP2	
Skin Swabs	Metagenomics	I4-FP5<br>I4-FP6	https://osdr.nasa.gov/bio/repo/data/studies/OSD-572/
	Metatranscriptomics	I4-FP5<br>I4-FP6	
Capsule Swabs	Metagenomics	I4-FP5<br>I4-FP6	https://osdr.nasa.gov/bio/repo/data/studies/OSD-573/

	Metatranscriptomics	I4-FP5<br>I4-FP6	
Skin Biopsy	Spatially Resolved Transcriptomics	I4-FP4	https://osdr.nasa.gov/bio/repo/data/studies/OSD-574/

Supplementary Table 4: Processed Data Map and Online Data Locations. Flight, recovery, and longitudinal profiles calculated for each data type and the link to the repository where they are housed.

Supplementary Table 5

Supplementary Table 5: cfRNA Calculations. Tissue of origin analysis from cfRNA sequencing. Tab 1 contains fractions of cell type specific RNA enrichment. Tab 2 contains comparisons between timepoints.

Supplementary Table 6

Supplementary Table 6: Recovery Profile Pathways. Overrepresented KEGG pathways during recovery from spaceflight in PBMCs. Tabs are split for CD4+ T cells, CD8+ T cells, CD14+ monocyte and CD16+ monocytes.

Supplementary Table 7

Supplementary Table 7: Metagenome and Metatranscriptome CVs. Species-level CV calculations across crew members for metagenomic and metatranscriptomic samples from oral, nasal, and skin swab samples.

Supplementary Table 8

Supplementary Table 8: Human Omics CVs. Gene/analyte-level CV calculations across crew members for NULISAseq, EVP proteomic, plasma proteomic, metabolomic, dRNA-seq and short read RNA-seq assays. GSEA pathway enrichment is calculated for pre-flight, post-flight (R+1), and recovery time intervals.

Supplementary Table 9

**Biospecimen**	**Assay(s)**	**OSDR Identifier**	**OSDR Link**
Whole Blood	• Oxford Nanopore Technologies Direct RNA-seq	OSD-569	https://osdr.nasa.gov/bio/repo/data/studies/OSD-569/

	• Quest Diagnostics Complete Blood Count (CBC)		
PBMC	• 10X Genomics Chromium Multiome Kit/ single-nuclei RNA-seq and ATAC-seq<br>• 10X Genomics Chromium T-cell repertoire (TCR) and B-cell repertoire (BCR) sequencing	OSD-570	https://osdr.nasa.gov/bio/repo/data/studies/OSD-570/
Blood Plasma	• Proteomics (Seer Proteograph)<br>• Proteomics of blood extracellular vesicles and particles<br>• Proteomic assay of blood plasma metabolome<br>• Element Biosciences cfDNA sequencing from blood plasma	OSD-571	https://osdr.nasa.gov/bio/repo/data/studies/OSD-571/
Crew Swabs (Oral, Nasal, Skin)	• Metagenomics<br>• Metatranscriptomics	OSD-572	https://osdr.nasa.gov/bio/repo/data/studies/OSD-572/
Environmental Swabs (Dragon Capsule)	• Metagenomics<br>• Metatranscriptomics	OSD-573	https://osdr.nasa.gov/bio/repo/data/studies/OSD-573/
Skin Biopsy	• NanoString GeoMx spatial transcriptomics<br>• NanoString GeoMx tissue images<br>• Deltoid swab metagenomics from biopsy site<br>• Deltoid swab metatranscriptomics from biopsy site	OSD-574	https://osdr.nasa.gov/bio/repo/data/studies/OSD-574/
Blood Serum	• Quest Diagnostics Comprehensive Metabolic Panel<br>• Alamar Bio and Eve Technologies Cytokine/Chemokine Biomarker Panel<br>• Eve Technologies Cardiovascular Biomarker Panel	OSD-575	https://osdr.nasa.gov/bio/repo/data/studies/OSD-575/
Stool	• Metagenomics	OSD-630	https://osdr.nasa.gov/bio/repo/data/studies/OSD-630/
Urine	• Alamar Bio	OSD-656	https://osdr.nasa.gov/bio/repo/data/stud

	Cytokine/Chemokine Biomarker Panel		ies/OSD-656/

Supplementary Table 9: Raw Data Online Locations. Raw data is located in the NASA Open Science Data Repository at the links in this table.

Supplementary Note 1

I4 Data Compendium: Wet Lab Methodology

Whole Genome Sequencing Extraction and Sequencing

DNA were extracted from the cell pellets of spun down cfDNA blood collection tubes (Streck, #230470) using the QIAamp Blood Maxi Kit (Qiagen #51192). The extracted DNA was shipped to Element Biosciences (San Diego, CA) for library preparation. The extracted DNA was quantified using Thermo Fisher Qubit dsDNA HS Assay Kit (cat# Q238253). KAPA HyperPrep Kit (Roche, cat# 07962363001) was used for preparing all extracted DNA samples per manufacturer's instructions. Adapters used were KAPA Unique-Dual Indexed Adapter Kit (Roche, cat# 08861919702). 8 DNA samples were library prepared, Covaris sheared and PCR-free. The DNA libraries were quantified using Thermo Fisher Qubit dsDNA HS Assay Kit (cat# Q238253) and sized using Agilent High Sensitivity DNA Kit (cat# 5067-4626).

The 8 DNA libraries generated with the KAPA HyperPrep Kit were processed using Adept Library Compatibility Kit (Element Biosciences, cat# 830-00003). Each library was circularized individually with an input of 0.5pmol (30 ul of 16.67nM). The final circularized libraries were quantified using qPCR standard and primer mix provided in the Adept Compatibility Workflow Kit (cat# 830-00007). The 8 circular libraries were pooled into 4 separate 2-plex pools. Each 2-plex pool was denatured and sequenced on Element AVITI system (Element Biosciences, Part #88-00001) using 2x150 paired end reads with indexing.

Clonal Hematopoiesis Profiling

Genomic DNA was obtained from the cell pellet of a cell-free DNA blood collection tube (Streck, cat# 230470) using the QIAamp Blood Maxi Kit (Qiagen, cat# 51192). Illumina sequencing adapters containing unique molecular identifiers were ligated onto short-read fragments according to the Kapa Bio-systems HyperPrep protocol (Roche, cat# 07962363001). Targeted enrichment of 9 genes (DNMT3A, TET2, ASXL1, TP53, CHEK2, JAK2, SRSF2, SF3B1, PPM1D) involved in clonal hematopoiesis was performed following the standard Invitae capture protocol. Deep sequencing was performed to a depth of ~ 15,000x using Novaseq 6000 and 150x150 S4 chemistry.

Biochemical and Blood Composition Measurements

Complete blood count (CBC) and comprehensive metabolic panels were completed by Quest Diagnostics. One 4mL tube of whole blood collected in a K2 EDTA tube (BD Biosciences, cat# 367844,) was used for the CBC, test code 6399. 500uL of serum from a serum separator tube (SST) (BD Biosciences, cat# 367987) was submitted for the CMP, test code 10231.

Serum samples were submitted to Eve Technologies (Calgary, Canada) for the biomarker profiling panels (1) Human Cytokine/Chemokine 71-Plex Discovery Assay® Array (HD71) and (2) Human Cardiovascular Disease Panel 3 9-Plex Discovery Assay® Array (HDCVD9). Concentration values were extrapolated using a 4 of 5 parameter logic standard curve. Measurements were normalized by average of pre-flight values from all crew.

Urine and serum samples were submitted to Alamar Biosciences (Fremont, CA) for the NULISA-seq Inflammation Panel, which targets 124 cytokines/chemokines and 80 other proteins related to immune response[1].

Direct RNA-Seq Library Preparation and Sequencing

Extracted RNA samples from PAXgene tubes (cat# 762165) were stored at -80C. Prior to library preparation, samples were thawed on ice. The Oxford Nanopore Technologies (ONT) direct RNA library prep protocol for PromethION was used (SQK-RNA002). Input amount for each sample library preparation was 500 ng of total RNA. Each final library was measured using Thermo Fisher Qubit 1X dsDNA HS Assay (cat# Q33231). Each library was divided in two, and brought up to loading volume by adding ONT's EB (Elution Buffer). One PromethION flow cell (FLO-PRO002) was used per library (i.e per sample), and libraries were sequenced on PromethION 48 beta for 72 hours. Runs were paused and the remaining half of each library was reloaded ~18 hours into the sequencing run onto their respective flow cells. Following this, runs were continued for the remainder of 72 hours.

Single-Cell Gene Expression, Chromatin Accessibility, and Immune Profiling

For each crew member, 8 mL of venous blood was collected in EDTA anticoagulant tubes (bD Biosciences, cat# 367844). Depletion of granulocytes was performed either directly from whole blood using the RosetteSep^{TM} granulocyte depletion cocktail (StemCell Technologies, cat# 15664) or by cell sorting after PBMC isolation. Whole blood was incubated in a granulocyte depletion cocktail (50 µL/mL of blood) for 20 minutes at room temperature. Next, Ficoll-Paque Plus (Cytiva, Sigma Cat#: GE17-1440-02) was utilized to isolate PBMCs by density gradient centrifugation. After washes in PBS with 2% FBS (Gibco, cat# 10082147) were completed, isolated PBMCs were cell sorted to remove granulocytes only if the RosetteSepTM granulocyte depletion cocktail was not added to whole blood prior to density gradient centrifugation. Granulocytes were identified using side scatter and the lymphocyte and monocyte fractions were sorted using a 100 µm nozzle (BD Aria). Following granulocyte depletion, PBMCs were split into two fractions to generate single cell V(D)J T-cell and B-cell libraries or multi-omic (GEX and ATAC) libraries. To capture T-cell and B-cell V(D)J repertoire, single cell gel beads-in-emulsion and libraries were performed according to the manufacturer's instructions (Chromium Next GEM Single Cell 5' v2, 10x Genomics). Prior to single cell multiome ATAC and gene

expression sequencing, nuclei isolation was performed by resuspending PBMCs in 100 µL of cold lysis buffer containing 10 mM Tris-HCl (pH 7.4), 10 mM NaCl, 3 mM MgCl2, 0.1 % Tween-20, 0.1 % Nonidet P40, 0.01 % digitonin, 1 % BSA, 1 mM DTT and 1 U/µL RNAse inhibitor. Cells were incubated for 4 minutes on ice, followed by the addition of 1 mL cold wash buffer (10 mM Tris-HCl (pH 7.4), 10 mM NaCl, 3 mM MgCl2, 0.1 % Tween-20, 1 % BSA, 0.1 % Tween-20, 1 mM DTT, 1 U/µl RNAse inhibitor). After centrifugation (500 RCF for 5 minutes at 4°C), nuclei were resuspended in diluted nuclei buffer (10X Genomics Single Cell Multiome ATAC kit A) at a concentration of 6,000 nuclei per µL. Single-nuclei libraries were generated via the Chromium Next GEM Single Cell Multiome ATAC and Gene Expression kit (10X Genomics) according to the manufacturer's instructions.

cfRNA Extraction and Sequencing

Plasma samples were collected in cfDNA Blood Collection (Streck: #230470) tubes at each timepoint and stored at -80C. Plasma samples were thawed at room temperature and subsequently centrifuged at 1300xg for 10 minutes at 4°C. cfRNA was isolated from the plasma supernatant (300-800uL) using the Norgen Plasma/Serum Circulating and Exosomal RNA Purification Mini Kit (Catalog No. 51000, Norgen). Next, 10 mL of DNase Turbo Buffer (Catalog No. AM2238, Invitrogen), 3 mL of DNase Turbo (Catalog No. AM2238, Invitrogen), and 1 mL of Baseline Zero DNase (Catalog No. DB0715K, Lucigen-Epicenter) was added to the extracted RNA and incubated for 30 minutes at 37°C. Subsequently, the treated RNA was concentrated into a final volume of 12 mL with the Zymo RNA Clean and Concentrate Kit (Catalog No. R1015, Zymo).

Sequencing libraries prepared from 8 uL of concentrated RNA using the Takara SMARTer Stranded Total RNA-Seq Kit v3 – Pico Input Mammalian (634485, Takara) and barcoded using the SMARTer RNA Unique Dual Index Kit (634451, Takara). Library concentration was quantified using a Qubit 3.0 Fluorometer (Q33216, Invitrogen) with the dsDNA HS Assay Kit (Q32854, Invitrogen). Libraries were quality-controlled using an Agilent Fragment Analyzer 5200 (M5310AA, Agilent) with the HS NGS Fragment kit (DNF-474-0500, Agilent). Libraries were pooled to equal concentrations and sequenced at the Cornell Genomics Core on an Illumina NextSeq 2000 machine using 150-base pair, paired-end sequencing for an average of 26 million reads per sample.

Plasma LC/MS Untargeted Proteomics

Sample preparation with the Proteograph™ Assay Protocol

Plasma was isolated from BD cell processing tubes (CPTs) containing sodium heparin (BD Biosciences, cat# 362753), stored at -80°C, then shipped on dry ice to Seer (Redwood City, CA) for sample processing and mass spectrometry-based proteomics analysis. Samples were processed with Seer Proteograph Assay[2,3] (cat# S55R1100). In brief: 250 µL from forty-two plasma samples were transferred to Seer Sample Tubes for processing with the Proteograph Assay kit (S55R1100). Plasma proteins were quantitatively captured in nanoparticle (NP)

associated protein coronas. To this end, 5 distinctly functionalized NP comprising superparamagnetic cores were employed. At equilibrium, NPs synergistically captured physicochemical subsets of the proteome and efficiently compress the high dynamic range of protein abundances to an accessible scale. The superparamagnetic core facilitates a fully automated liquid handling process. Proteins were subsequently denatured, reduced, alkylated and subjected to proteolytic digestion (trypsin and Lysc). Peptides were purified and yields were determined (Thermo Fisher Scientific cat # 23290). Peptides were dried down overnight with a vacuum concentrator and reconstituted with a reconstitution buffer to a concentration of 25 or 50 ng/µL, depending on nanoparticle (NP) peptide preparation.

Data-Independent Acquisition LC-MS/MS

For Data-Independent Acquisition (DIA), 4 µL of reconstituted peptide mixture from each NP preparation was analyzed resulting in a constant mass MS injection between samples (200 ng: NP1, NP2, NP3, and NP5; 100 ng: NP4). Each sample was analyzed with an UltiMate3000 nanoLC system coupled with a Bruker timsTOF Pro mass spectrometer using a trap-and-elute configuration. First, the peptides were loaded onto an Acclaim^{TM} PepMap^{TM} 100 C18 (0.3 mm ID x 5 mm) trap column and then separated on a 50 cm µPAC^{TM} analytical column (PharmaFluidics, Belgium) at a flow rate of 1 µL/min using a gradient of 5 – 25% solvent B (0.1% formic acid, 100 % acetonitrile) mixed into solvent A (0.1% formic acid, 100% water) over 22 min, resulting in a 33 min total run time. The mass spectrometer was operated in diaPASEF mode using ion mobility range of 0.57 – 1.47 V*s/cm2, TIMS Ramp of 100 ms and 100 ms TIMS accumulation time. Scan ranges were 100-1700 m/z (MS1) and 400-1000 m/z (MS2). One-hundred fixed window MS2 DIA scans were collected per cycle.

Plasma-derived Extracellular Vesicle and Particle (EVP) Characterization

Blood pre-processing for EVP isolation

4mL of whole blood was collected into EDTA tube(s) (BD Biosciences, cat# 367844) and shipped overnight on ice to Weill Cornell Medicine (New York, NY). Blood was centrifuged at 500 x g for 10 minutes, then plasma was transferred to a new tube and centrifuged at 3000 x g for 20 minutes, and the supernatant was collected and stored at -80°C for EVP isolation. Plasma volumes ranged between 0.6 - 1.7 ml.

EVP isolation by sequential ultracentrifugation

Plasma samples were thawed on ice and EVPs were isolated by sequential ultracentrifugation, as previously described[4]. Briefly, samples were centrifuged at 12,000 x g for 20 minutes to remove microvesicles, then EVPs were collected by ultracentrifugation in a Beckman Coulter Optima XE or XPE ultracentrifuge at 100,000 x g for 70 minutes. EVPs were then washed in PBS and pelleted again by ultracentrifugation at 100,000 x g for 70 minutes. The final EVP pellet was

resuspended in PBS and protein concentration was measured by Pierce BCA Protein Assay (Pierce, Thermo Fisher Scientific).

Nanoparticle tracking analysis (NTA) of plasma EVPs

Plasma depleted of large microvesicles (post 12,000 x g centrifugation step) was diluted 400-fold in PBS and EVP size and particle number were characterized by nanoparticle tracking analysis (NTA) using the DS500 nanoparticle characterization system (NanoSight, Malvern Instruments) equipped with a violet laser (405 nm).

Transmission electron microscopy (TEM)

For negative staining TEM analysis, 0.1 mg/ml of EVPs in PBS were placed on a formvar/carbon coated grid and allowed to settle for 1 min, as previously described[4]. The sample was blotted and negatively stained with 4 successive drops of 1.5% (aqu) uranyl acetate, blotting between each drop and after the last drop of stain, the grid was blotted and air-dried. Grids were imaged with a JEOL JSM 1400 (JEOL, USA, Ltd, Peabody, MA) transmission electron microscope operating at 100Kv. Images were captured on a Veleta 2K x 2K CCD camera (Olympus-SIS, Munich, Germany).

Western blot

We validated human plasma-derived EVP markers by immunoblotting. For this, 5 μg of EVP protein were subjected to denaturing electrophoresis (SDS-PAGE), transferred onto a PVDF membrane and incubated overnight at 4°C with primary antibodies against human CD9 (rabbit monoclonal IgG, 1:1000 dilution, 13174S, Cell Signaling), Galectin 3 Binding Protein (LGALS3BP) (mouse monoclonal IgG, 1:1000 dilution, sc-374541, Santa Cruz Biotechnology), Fibronectin (FN1) (rabbit polyclonal IgG, 1:1000 dilution, ab2413, Abcam), and for β-actin (rabbit monoclonal IgG, 1:1000 dilution, 8457S, Cell Signaling). Secondary antibodies were horseradish peroxidase (HRP)-labeled IgG goat anti-rabbit or goat anti-mouse (1h incubation, 1:5000 dilution, Jackson Laboratory). All antibodies were diluted in 5% BSA in Tris-buffered saline with 0.1% Tween-20 (Thermo Fisher Scientific). The immunocomplexes were developed using the SuperSignal™ Western Blot Enhancer (Pierce, Thermo Fisher Scientific) and the membranes were imaged using a ChemiDoc Imaging System (Bio-Rad).

Proteomic mass spectrometry analyses

Enriched EVP samples (2 μg - adjusted based on BCA measurements) were processed as previously described [4]. Briefly, samples were dried by vacuum centrifugation and re-dissolved in 30-50 μL 8M Urea/50 mM ammonium bicarbonate/10 mM DTT. Following lysis and reduction, proteins were alkylated using 20 or 30 mM iodoacetamide (Sigma). Proteins were digested with Endopeptidase Lys C (Wako) in < 4M urea followed by trypsinization (Promega) in < 2M Urea. Peptides were desalted and concentrated using Empore C18-based solid phase extraction prior to analysis by high resolution/high mass accuracy reversed phase (C18) nano-LC-MS/MS.

Typically, 30% of each sample was injected. Peptides were separated on a pulled-emitter column using a 90-minute gradient increasing from 2 to 30% buffer B/buffer A (buffer A: 0.1% formic acid in water, buffer B: 0.1% formic acid in 80% acetonitrile). Samples were analyzed on Q-Exactive HF LC-MS/MS instrument (Thermo Scientific) operated in data dependent (DDA) positive ion mode.

Plasma LC-MS/MS Untargeted Metabolomics

Sample Preparation and Metabolite Extraction

To survey a broad range of potential changes in plasma metabolite levels, acquired plasma were extracted for LC/MS-based metabolite profiling using a combination of aqueous normal phase (ANP) and reverse phase (RP) chromatographic separations, followed by positive- and negative-ion MS analysis. For ANP LC/MS metabolite profiling, plasma metabolites were extracted by addition of 1 parts of plasma to 20 parts 70% acetonitrile in ddH_2O (vol:vol). The mixture was briefly vortexed and then centrifuged for 5 min at 16,000 x g to pellet precipitated matter. The resulting supernatant was transferred to sample vials and 4 µl was injected for ANP LC/MS metabolite profiling. For RP LC/MS metabolite profiling, extraction of plasma was carried out using -70°C cold methanol, methyl tert-butyl ether (MTBE), and water. For this purpose, 8 µL of plasma was added to 180 µL of the cold methanolic extraction media in a 1.5 mL Eppendorf tube and briefly vortexed. This was followed by addition of 600µL ice-cold MTBE to the tube, with intermittent vortex and shaking on ice for 6 min. Phase separation was induced by adding 150 µl of LC-MS-grade H_2O to the methanol/MTBE extracts. The final mixture was vortexed again, followed by centrifugation at 14,000 rpm for 2 min at 4°C. The upper phase was transferred to clean tubes, dried down and stored at -80°C until the day of analysis. On the day of LC/MS analysis, dried down metabolites were resolubilized in 100 µl of a solution consisting of 36% acetonitrile, 36% isopropyl alcohol, 28% H_2O, 0.1% formic acid, and 5 mM ammonium formate (i.e., 3:2 volume ratio of LC mobile phases A and B). The resulting reconstitute was briefly vortexed and centrifuged for 2 min at 14,000 rpm. Supernatants were transferred to LC sample vials and a 4 µl volume was injected for RP LC/MS analysis using both positive and negative ion MS detection modes as detailed below. Plasma extracts for ANP analysis was performed as described previously[5–7].

LC/MS of the ANP and RP of Plasma Metabolites

LC/MS analysis of sample extracts was performed using a platform comprised of an Agilent Model 1290 liquid chromatography system coupled to an Agilent 6550 *iFunne*l time-of-flight MS analyzer[5–7]. Chromatographic separation of metabolites was performed using both ANP and RP gradient separations, as noted above. For ANP chromatography, plasma metabolites were separated on a Diamond Hydride column (4 µm, 100A, 150 mm X 21 mm ID; MicroSolv Technology Corp.). Mobile phases consisted of: (A) 50% isopropanol, containing 0.025% acetic acid, and (B) 90% acetonitrile containing 5 mM ammonium acetate. To eliminate the interference of metal ions on chromatographic peak integrity and electrospray ionization, EDTA

was added to the mobile phase at a final concentration of 5 μM. The following gradient was applied: 0–1.0 min, 99% B; 1.0–20.0 min, to 0%B; 20.0 to 29.0, 0% B; 29.1 to 37 min, 99%B. The flow rate was 0.3 ml/min and column temperature was maintained at 25°C. For RP chromatography, plasma lipids were preferentially separated using an Agilent Zorbax Eclipse Plus C18 column(1.8 μm, 100 × 2.1 mm ID, Agilent Technologies). Mobile phases consisted of: (A) 60:40 (v/v) acetonitrile:water containing 10 mM ammonium formate and 0.1 % formic acid, and (B) 90:10 (v/v) isopropanol:acetonitrile containing 10 mM ammonium formate and 0.1 % formic acid. The following gradient was applied: 0 min: 15% B; 0–2 min to 30% B, 2–2.5 min to 48% B, 2.5–8.5 min to 72% B, 8.5–11.5 min to 99% B,11.5–12 min to 99% B, 12.1–15 min 15% B. The flow rate was 0.6 ml/min with a column temperature of 60°C. To eliminate potential artifacts owing to LC/MS instrument drift, metabolite instability, and other experimental factors that may contribute to systematic error, the sample run sequence was arranged such that no two consecutively analyzed samples came from either the same patient or were from the same blood sampling date.

cfDNA Extraction and Sequencing

cfDNA was isolated from 500uL aliquots of plasma from cfDNA blood collection tubes (Streck, #230470). Two 500uL tubes per sample were thawed on ice prior to processing. cfDNA was extracted from each crew member from all time points simultaneously, totaling to 24 extractions – 4 crew members from 6 time points. cfDNA was extracted using Qiagen's QIAamp ccf DNA/RNA Kit (cat# 55204) per manufacturer's instructions and eluted in 15 uL Qiagen elution buffer per sample. Yield was measured for each sample using Thermo Fisher Qubit 1X dsDNA HS Assay (cat# Q33231).

Entire extract volume was used as input for library preparation using NEBNext Ultra II DNA Library Preparation Kit (cat# E7645L) for cfDNA protocol, which skips the fragmentation step. Each sample was barcoded using NEBNext Multiplex Oligos for Illumina (Unique Dual Index UMI Adaptors - 96 reactions, cat# E6440S) per manufacturer's instructions. Final library was eluted in 30uL and checked for concentration using Thermo Fisher Qubit 1X dsDNA HS Assay (cat# Q33231). Fragment sizes were determined using Agilent's Tapestation 2100 and D1000 reagents and screentape (cat# 5067-5584, 5067-5585). Fragment size average was at ~380 bp. 0.25 pmol of each sample was then shipped to Element Biosciences (San Diego, CA) for sequencing.

A total of 24 cfDNA libraries generated with the NEBNext Ultra II DNA Library Preparation kit were processed using Adept Library Compatibility Kit (Element Biosciences, cat# 830-00003). Each library was circularized individually with an input range of 0.2-0.5 pmol (30 ul of 6.67-16.67nM) based on linear library yields. The final circularized libraries were quantified using qPCR standard and primer mix provided in the Adept Library Compatibility Kit (v1.1). The 24 circular libraries were pooled into 2 separate 4-plex pools. Each 4-plex pool was denatured and

sequenced on Element AVITI system (Element Biosciences, Part #88-00001) using 2x147 paired reads with 19bp UMI/index 1 and 8 bp index 2. Primary analysis was performed onboard the AVITI sequencing instrument (cat# 880-00001).

Telomere length assessment by multiplexed quantitative (q)PCR

Blood samples were prepared for processing by cutting three 3 mm circular punches from a Whatman™ 903 Protein Saver Card (cat# WHA10534612) with the collected blood samples using an Integra™ Miltex™ Standard Biopsy Punch (cat# 12-460-406) and placed into a 1.5 ml microcentrifuge tube using a sterile pair of tweezers. Samples were prepared using the Qiagen QIAamp DNA Investigator Kit (cat# 56504) following the manufacturer's protocol for Isolation of Total DNA from FTA and Guthrie Cards. Following the completion of the extraction protocol, the extracted DNA was quantified using Thermo Fisher Qubit 1X dsDNA HS Assay (cat# Q33231) following the manufacturer's protocol.

DNA was shipped to Colorado State University (Fort Collins, CO) where multiplexed qPCR measurements of average telomere length were carried out as previously described[8]. Here, a 22 µL master mix was prepared using SYBR green GoTaq qPCR master mix (Promega #A6001) combined with the telomere forward primer (TelG; 5'-ACACTAAGGTTTGGGTTTGGGTTTGGGTTTGGGTTAGTGT-3'), telomere reverse primer (TelC; 5'-TGTTAGGTATCCCTATCCCTATCCCTATCCCTATCCCT AACA-3'), albumin forward primer (AlbU; 5'-CGGCGGCGGGCGGCGCGGGCTGGGCGGA AATGCTGCACAGAATCCTTG-3'), albumin reverse primer (AlbD; 5'-GCCCGGCCCGCCG 4 CGCCCGTCCCGCCGGAAAAGCATGGTCGCCTGTT-3') at 10 µM per primer (Integrated DNA Technologies), and RNase/DNase free water. To the master mix, 3 µL of DNA at 3.33 ng/uL was added for a final volume of 25 µL. The TelG/C primers were at a final concentration of 900 nM and the AlbU/D primers at 400 nM. Telomere length measurements were carried out using a Bio-Rad CFX-96 qPCR machine. The cycle design was as follows: 95°C for 3 min; 94°C for 15 s, 49°C for 15 s, for 2 cycles; 94°C for 15 s, 62°C for 10 s, 74°C for 15 s, 84°C for 10 s, and 88°C for 15 s, for 32 cycles. The melting curve was established by a 72°C to 95°C ramp at 0.5°C/second increase with a 30 second hold. Multiplexing both telomere and albumin primers using a single fluorescent DNA-intercalating dye is possible because the telomere sequences are amplified at a lower quantification cycle (Cq) than the albumin sequences. Standard curves were prepared using human genomic DNA (Promega, cat # G3041) with 3-fold dilutions ranging from 50 ng to 0.617 ng in 3 µL per dilution. Negative controls included a no-template TelG/C only and AlbU/D only, and a combined TelG/C and AlbU/D control. Samples were normalized across plates using a human genomic DNA standard. Each sample was run in triplicate on a 96-well plate format and relative telomere length was established using a telomere (T) to albumin (A) ratio.

Spatially Resolved Transcriptomics of Skin Tissue

Skin biopsy samples from 4 crew members were collected and frozen in cryovials pre and post flight. Samples were mailed to NanoString for profiling on the GeoMx DSP (Seattle, WA). Collected skin was flash embedded in OCT blocks. Three or 4 replicate OCT-embedded tissues were placed on a single slide per patient including pre and post flight replicates. Immunofluorescent visualization marker for Pan-Cytokeratin (PanCK, Novus cat# NBP2-33200, Alexa Fluor® 532, clone ID AE1 + AE3), fibroblast activation protein (FAP, Abcam cat# ab222924, Alexa Fluor® 594, clone ID EPR20021) and smooth muscle actin (SMA, R&D Systems cat# IC1420R, Alexa Fluor® 647, clone 1A4) were used for region or interest (ROI) selection. The DSP whole transcriptome assay (WTA) was used to assess genes collected in each ROI. For DSP processing, OCT slides were thawed overnight in 10% NBF at 4°C followed by PBS washes for thorough fixation. After washes, slides were prepared following the automated Leica Bond RNA Slide Preparation Protocol (Leica, 21.2821) for fixed frozen samples, digesting samples with 1.0 µg/mL protease K for 15 min, and antigen retrieval for 20 min at 100°C (NanoString, no. MAN-10115-05). In situ hybridizations with the GeoMx Whole Transcriptome Atlas Panel (NanoString, WTA - 18,677 genes, cat# GMX-RNA-NGSHuWTA-4) at 4 nM final concentration were done in Buffer R (NanoString, GMX-PREP-RNAFFPE-12). Morphology markers were prepared for four slides concurrently using Syto13 (DNA), PanCK, FAP and SMA in Buffer W for a total volume of 225 µl per slide. Slides incubated with 225 µl of morphology marker solution at RT for 1 h, then washed in SSC and loaded onto the NanoString DSP instrument. The 20x scan was used to select freeform ROIs to guide selection of outer epidermal (OE), inner epidermal (IE), outer dermal (OD) and vascular (VA) regions. ROI libraries were sequenced on a NovaSeq 6000.

Oral, Nasal, and Skin Swab Nucleic Acid Extraction

Samples were collected prior to processing as isohelix swabs stored in 400 ul of Zymo Research DNA/RNA shield (cat# R1100) in Thermo Fisher Matrix 1.0 mL ScrewTop Tubes (cat# 3741-WP1D-BR). Prior to beginning the extraction protocol, samples were vortexed for 5 seconds using the MO BIO Vortex Adapter tube holder (cat# 13000-V1-24) to ensure maximum biomolecular yield from the collected swabs. DNA/RNA extraction was performed using the Qiagen Allprep Bacterial DNA/RNA/Protein Kit (cat# 47054). Steps one and two were omitted from the Qiagen Allprep Bacterial DNA/RNA/Protein Kit manufacturer's protocol as these samples were not generated through bacterial culture. Aside from this omission, samples were extracted following the manufacturer's protocol. Following the completion of the extraction protocol, the extracted DNA was quantified using Thermo Fisher Qubit 1X dsDNA HS Assay (cat# Q33231), extracted RNA was quantified using Thermo Fisher Qubit RNA HS Assay Kit (cat# Q32855), and extracted protein was measured using the Thermo Fisher Rapid Gold BCA Protein Assay (cat # A53225). All quantification protocols were conducted per manufacturer’s instructions.

Metagenome and Metatranscriptome Sequencing

Metagenome Library Preparation and Sequencing

Illumina DNA Library prep kit (cat# 20060059) was used for preparing all extracted DNA samples for Illumina Whole genome sequencing per manufacturer's instructions. Adapters used were IDT® for Illumina® DNA/RNA UD Indexes, Tagmentation (96 Indexes, 96 Samples). 408 DNA samples were library prepared and pooled into a total of 4 pools. Each library pool was sequenced using 2 X 150 Paired end sequencing on S4 flow cell using NovaSeq 6000 sequencer, one pool per lane of the S4 flow cell.

A subset of library pools were also sequenced on the AVITI sequencing instrument (cat# 880-00001). Two pools of 96 indexed metagenome libraries prepared above, were processed using Adept Library Compatibility Kit (Element Biosciences, cat# 830-00003). Each library pool was circularized individually with an input of 0.5pmol (30 ul of 16.67nM). The final circularized library pools were quantified using qPCR standard and primer mix provided in the Adept Library Compatibility Kit. Each library pool was denatured and sequenced on Element AVITI system (Element Biosciences, Part #88-00001) using 2x150 paired end reads with indexing, to a depth of at least 20M read-pairs for each library. Primary analysis was performed onboard the AVITI sequencing instrument. FASTQ files were analyzed using a secondary analysis pipeline from Sentieon.

Metatranscriptome Library Preparation and Sequencing

For library preparation, total RNA samples were sent to Hudson Alpha (Huntsville, AL). Samples were treated with DNAse 1 (Zymo Research, cat# E1010). Post-DNAse digested samples were then put into the NEBNext rRNA depletion v2 (Human/Mouse/Rat), Ultra II Directional RNA (10 ng), and Unique Dual Index Primer Pairs were used following the vendor protocols from New England Biolabs. Completed libraries were quantified by Qubit or equivalent and run on a Bioanalyzer or equivalent for size determination. Libraries were pooled and quantified by Qubit fluorometer (ThermoFisher Scientific), TapeStation 2200 (Agilent), and qRT-PCR using the Kapa Biosystems Illumina library quantification kit.

Cut&Run Sample Collection, Library Preparation, and Sequencing

Whole blood was collected from a K2 EDTA tube at the R+194 time point. T cells were isolated using the MACSxpress Whole Blood Cell Isolation cocktail according to the manufacturer's protocol (Miltenyi Biotec, #130-098-193). Red Blood Cell Lysis Solution was used to remove any remaining red blood cells in the cell suspension (Miltenyi Biotec, #130-094-183). Cells were pelleted at 300×g for 10 minutes at room temperature and resuspended in freezing media (DMSO(10%)/FBS(90%) solution). Cells were frozen until the time of the Cut&Run protocol.

Cut&Run libraries for C002 crew member T cells were prepared following the Epicypher Cutana Cut&Run protocol adapted for a 96 well plate format. All reagents and spin columns were obtained from CUTANA ChIC/CUT&RUN kit (SKU: 14-1048). 10,000 cells were washed and bound with activated ConA beads. After magnetic separation, supernatant was removed and antibody buffer solution was added to each sample along with 1:100 dilutions of Invitrogen antibodies for Abcam H3K27ac (CAT:ab245911) and Epicypher H3K4me1 (SKU:13-0040). Cutana Rabbit IgG antibody (SKU: 13-0042) was used as a negative control. Samples were incubated overnight at 4°C rotating followed by magnetic separation and washing with 0.01% digitonin buffer. Cutana pAG-MNase were bound to each sample washed again twice with digitonin buffer and chromatin was digested and released with 100 mM $CaCl_2$ and incubated for 2 hrs at 4°C. Stop buffer with 1ng of Cutana E.coli Spike-in DNA (SKU: 18-1401) was added to each sample followed by an incubation at 37°C for 10 mins. 10ul of purified DNA fragments were used for library prep following the NEBNext Ultra II for DNA protocol but with 16 amplification cycles. Samples were loaded on a Novaseq S4 flow cell and sequenced paired end 50x50 at 40 million reads.

I4 Data Compendium: Dry Lab Methodology

WGS and cfDNA Data Processing

Primary analysis was performed onboard the AVITI sequencing instrument. Resultant FASTQ files were validated using FastQC (v0.11.9) and MultiQC (v1.13). Read adapters were trimmed at 3' and 5' ends for low quality using Trim Galore (v0.6.5), lower quality reads were classified and removed, retaining only those reads with length >= 25bp, and phread quality >= 20. Reads were aligned against the hg38 human reference genome with BWA MEM (v.0.7.15) and subjected to standard QC and deduplication procedures as a part of Sentieon's TNscope[9] (v202010) DNAseq workflow.

Clonal Hematopoiesis Data Processing

UMI Consensus was built using Sentieon's UMI extract tool, alignment to the GRCh 38 reference genome was performed with BWA MEM (v0.7.15). Sentieon TNscope RNA-seq variant pipeline (v202010) was used for variant calling, filtering of reads based on mapping quality, depth, and strand bias[9]. BCFtools[10] (v1.9) was used to filter by triallelic sites, short tandem repeats, read quality and read position bias. Varient-Effect-Predictor VEP[11] (v107) and SnpEff[12] (v4.3) was used for annotation of variants and further filtering based on predicted impact of mutations. Data wrangling, tidying, and visualizations were performed using R (v4.1.2), RStudio (v2021.09.2) and libraries (Tidyverse, Dplyr, data.table, ggplot2).

Single-Cell Gene Expression, Chromatin Accessibility, and Immune Profiling

The resulting single-nuclei GEX and single-nuclei ATAC-seq FASTQ files were aligned using the cellranger-arc pipeline (v2.0.0) from 10X genomics against human reference genome hg38. We followed the 10X single-cell multi-ome analysis pipeline as previously reported and adapted for this data as described in Barisic *et al*. (https://doi.org/10.1016/j.ccell.2024.02.010). Quality control and cell annotation was performed on the snATAC seq peak-cell matrices, and the snGEX gene-cell matrices using the R Seurat package (version 4.2.0)[13]. Subpopulations were clustered and labeled using a publicly available Azimuth human PBMCs reference[14] in conjunction with Seurat's supervised clustering functionality. Differential gene expression analysis for labeled subpopulations was performed using the FindMarkers functionality of Seurat, with a log fold change (logFC) cutoff point of 0.25. P-values of resultant genes were measured using the Wilcoxon Rank Sum test and deemed significant for $p < 0.05$. Differentially accessible regions for the labeled subpopulations were identified using the same method. VDJ libraries were processed using Cell Ranger v6.1.1. Reads were aligned to the GRCh38 human genome. TCR and BCR annotated data were analyzed with the VGenes package (https://github.com/WilsonImmunologyLab/VGenes).

Direct RNA-seq Data Processing

Fast5 files were basecalled with Guppy version 6.2.1 [community.nanoporetech.com], and alignment of raw nanopore events to the basecalled sequences was done with the f5c module eventalign (v1.1)[15]. Quality assessment was performed with pycoQC (v2.5.0.21)[16] and MultiQC (v1.13)[17]. The Oxford Nanopore Technologies pipeline, pipeline-transcriptome-de[18], was used to quantify transcripts and to subsequently perform the differential expression analysis. pipeline-transcriptome-de performs the quantification with salmon[19], accounting for multi-mapping reads; therefore, we included the highest-scoring secondary minimap2 alignments (minimap2 switch -p1.0) in the input for the pipeline. Differential gene expression results, which pipeline-transcriptome-de generates with edgeR[20] while collapsing transcripts into respective genes. Differentially expressed genes were also calculated using minimap2 (v2.24-r1122)[21], featureCounts (v2.0.1)[22], DESeq2 (v1.36.0)[23], and the SARTools (v1.8.1)[24] wrapper package. m6Anet (v)[25] was used to detect m6A modifications. As required by m6Anet, the transcriptomic alignments were filtered to retain primary mappings only (minimap2 switch --secondary=no). Sites of m^6A modifications were annotated with probabilities of methylation via m6Anet, and methylKit (v3.18)[26] was then used to discover differentially methylated sites between conditions; sites were deemed significant if the resultant methylKit q-value fell below 0.01, as recommended by the tool's authors.

cfRNA Data Processing

The cfRNA sequencing data was processed using a custom bioinformatics pipeline utilizing the Snakemake workflow management system (v7.7.0). Reads were quality filtered and trimmed using BBDUK (v38.90), aligned to the Gencode GRCh38 human reference genome (v38,

primary assembly) using STAR (v2.7.0f) default parameters, deduplicated using UMI tools (v1.1.2), and features quantified using featureCount (v2.0.0). Mitochondrial, ribosomal, X, and Y chromosome genes were removed prior to analysis. cfRNA sample quality was determined by calculating DNA contamination (intron/exon ratio), rRNA contamination, number of feature counts, and RNA degradation. All samples passed QC. Read counts of technical duplicate samples were combined for downstream analyses.

Cell type deconvolution was performed using BayesPrism (v2.0) with the Tabula Sapiens single cell RNA-seq atlas (Release 1)[74,75]. Cells from the Tabula Sapiens atlas were grouped as previously described in Vorperian et al.[76].

Comparative analysis of DEGs was performed using a negative binomial model as implemented in the DESeq2 package (v1.34.0) using a Benjamini-Hochberg corrected p value cutoff <0.05, unless otherwise stated. Variance stabilization transformation was used for comparing and plotting gene counts, unless otherwise stated.

Plasma Proteomic Data Processing

DIA Raw Data Processing

DIA data was processed using Proteograph™ Analysis Suite (PAS). Raw MS data was processed using the DIA-NN search engine (version 1.8), searching MS/MS spectra against human protein entries (UP000005640_9606) and a human plasma spectral library generated from DDA runs (62,687 precursors and 4,011 protein groups). MS1 and MS2 mass accuracy was set to 10 ppm. Precursor FDR was set to 0.01, and PG Q value was set to 0.01. Quantification was performed on summed abundances of all unique peptides considering only precursors passing the q-value cutoff. PAS summarizes all nanoparticle values for a single protein into a single quantitative value. Specifically, a single protein may have been measured up to five times, once for each nanoparticle. To derive the single measurement value, PAS uses a maximum representation approach, whereby the single quantification value for a particular peptide or protein group represents the quantitation value of the NP most frequently measured across all samples.

Downstream Statistical Analysis of Plasma Proteomics

Technical duplicates (2 per sample) were first averaged and the average was used for downstream analysis. Protein groups were filtered by coefficient of variance (CV), keeping only proteins with CV<0.5 in at least one time point and by data sparsity, keeping proteins that have no NAs in with at least one condition. The filtered data was normalized with "vsn" normalization[27,28] and imputed with random draws from a Gaussian distribution centered around a minimal value[28]. Differential gene expression analysis was performed with *"limma"* R package (version 3.52)[29], taking into account estimated sample weights[30] and including astronaut as a factor in the model. Heatmaps of significant proteins (adj p.value <0.05 and logFC| >1) from the different comparisons performed were plotted with *"pheatmap"* (version 1.0).

EVP Proteomic Data Processing

Proteomic database search

High resolution/high mass accuracy nano-LC-MS/MS data was processed using Proteome Discoverer 1.4.1.14 (Thermo-Scientific, 2012)/ Mascot 2.5[31], as previously reported[4]. Human data was queried against the UniProt's Complete HUMAN proteome (February, 2020: 74,790 sequences) using the following parameters: Enzyme: Trypsin/P, maximum allowed missed cleavage sites: 2, monoisotopic precursor mass tolerance: 10 ppm, monoisotopic fragment mass tolerance: 0.02 Da, dynamic modifications: Oxidation (M), Acetyl (Protein N-term), static modification: Carbamidomethyl (C). Percolator (v3.6.2) was used to calculate peptide False Discovery Rates (FDR), which was calculated per file. A false discovery rate (FDR) of 1% was applied to each separate LC-MS/MS file. For plasma, the sequences of porcine trypsin and Endopeptidase LysC were concatenated to the human databases.

Downstream Statistical Analysis of EVP Proteomics Data

Downstream analysis was performed in the same way as for the plasma proteomics mentioned above (with the exception that there were no technical duplicates). Heatmaps of significant proteins (adj p.value <0.05 and logFC| >1) from the different comparisons performed were plotted with *"pheatmap"* (v 1.0). To perform tissue of origin deconvolution of the exosomes, a list of proteins "enriched" or "specific" to 36 different tissues compiled from the Human Protein Atlas (https://www.proteinatlas.org/)[32] was used as pathway input to *fgsea* (version 1.22) with a minimum size=5 and maximum size=500. The ranked list of EVP proteins from the (R+1 vs Pre-Flight) comparison was used as gene list input.

Metabolomics Data Processing

Raw Data Processing of Plasma Metabolites

Raw LC/MS data were extracted using MassProfinder 8.0 (Agilent Technologies), employing an in-house annotated personal metabolite database comprising 865 metabolites for ANP chromatography and 270 lipid metabolite RP database curated from the Agilent plasma lipidomic database (https://www.agilent.com/cs/library/applications/application-LCMS-Plasma-Lipid-Analysis-Method-ZORBAX-Jet-Stream-5991-9280EN-agilent.pdf.pdf). Metabolite identities were inferred based on accurate mass and retention time matches of database. Confidence levels for metabolite annotations were indicated on a 1-4 scale, in accord with guidelines from the Lipidomics Standard Initiative (https://lipidomics-standards-initiative.org/). Relative abundance of extracted metabolites in units of *ion counts* was exported for downstream statistical analysis.

Downstream Statistical Analysis of Plasma Metabolite Data

Metabolites with over 20% missing values and samples with over 10% missing values were filtered out. We performed probabilistic quotient normalization to correct for sample-wise

variation and then log2 scaled the data. To remove outliers, we removed measurements with <2.5% two-tailed probability to originate from the same normal distribution as the rest of the measurement values, after applying a Bonferroni-inspired correction factor (division by sample size). Metabolites with over 20% missing values were filtered again, and missing values were then imputed using a k-nearest-neighbor algorithm. After processing, "limma" was used for group comparisons with the same design as the proteomics. Heatmaps of significant proteins (adj p.value <0.05 and logFC| >1) from the different comparisons performed were plotted with *"pheatmap"* (v 1.0).

Spatially Resolved Transcriptomics of Skin Tissue Data Processing

GeoMx WTA sequencing reads from NovaSeq6000 were compiled into FASTQ files corresponding to each ROI. FASTQ files were then converted to digital count conversion files using the NanoString GeoMx NGS Pipeline (v2.3.4). From the normalized count matrix, DESeq2 (v1.34.0) was used to perform differential expression analysis, and FGSEA[33] (v1.20.0) was used for pathway enrichment analysis. GSVA[34] (v1.42.0) was used to run ssGSEA analysis with custom gene signatures obtained from published single cell and bulk skin datasets.

Metagenome and Metatranscriptome Data Processing

Sample quality control

Metagenomic and metatranscriptomic samples were subjected to identical quality control (QC) checks before further analytic processing. All tools and software were configured and executed using default settings, unless noted otherwise. Samples were ingested into the QC pipeline using bbtools (V38.92), where clumpify [parameters: optical=f, dupesubs=2,dedupe=t] would group the reads, bbduk [parameters: qout=33 trd=t hdist=1 k=27 ktrim="r" mink=8 overwrite=true trimq=10 qtrim='rl' threads=10 minlength=51 maxns=-1 minbasefrequency=0.05 ecco=f] acted to remove contaminating adapters, and lastly tadpole [parameters: mode=correct, ecc=t, ecco=t] to account for sequencing error.[35] bbtool's repair functionality was used to remove any remaining unmatched reads. Lastly, samples were aligned to the human genome using Bowtie2 (parameters: --very-sensitive-local, v2.2.3) in order to remove potentially remaining human-contaminating reads.[36]

Taxonomic abundance quantification

Metagenomic and metatranscriptomic samples were assessed for bacterial and viral taxonomic composition using a variety of approaches. XTree (https://github.com/GabeAl/UTree) [parameters: –redistribute]. A more recently updated version of Utree[37], was used to generate all figures found in the main text of the paper. XTree functions as a k-mer based aligner (similar to Kraken2[38], but designed to work more efficiently using larger bases). It utilizes capitalist read redistribution[39] to choose the likeliest map between a given reference and a read, determined based on the strength of association of all reads for that specific reference. This process generates the aggregate coverage of a given query genome, along with a report of regions found only within one

genome in the entire database, called “total unique coverage”.

Bacterial alignments were performed using a k-mer database generated by Xtree [parameters: xtree BUILD k 29 comp 0] from the Genome Taxonomy Database representative species dataset (Release 207). Allowing for the alignment of metagenomic and metatranscriptomic samples. Viral alignments were performed using a separate Xtree database [parameters: xtree BUILD k 17 comp 0] from all complete GenBank viral genomes. The sequences were de-replicated using a BLAST 99% identity threshold, via published approaches (https://github.com/snayfach/MGV/tree/master/ani_cluster).[40,41]

All Xtree alignments were then filtered with multiple parameters for total and unique coverage reflected in the filenames of the resource. See below:

For bacterial databases:

1) 5% total and 0.25% unique
2) 1% total and 0.5% unique
3) 10% total and 5% unique

For viral databases:

1) 10% total and 5% unique
2) 1% total and 0.5% unique

Additionally, both kraken2 and bracken were utilized as an alternate means of measuring taxonomic composition of our samples (using factory settings), where kracken2 called the taxa, and bracken quantified their given abundance[38,42]. This was done using the default reference databases for kraken2. For the data reported in Figure 6E-F, kraken2 taxa were filtered by the condition that they should have a prevalence of at least 25 samples, where at least one of the samples should have at least 500 reads that could align to a given organism.

Alpha and beta diversity metrics were computed for measured taxonomic abundance using the vegan package (v2.6.4) in R[43].

Metagenomic assembly, bacterial/viral binning, and bin abundance quantification

All samples were assembled using MetaSPAdes V3.14.3 (--assembler-only).[44] Assembly quality was measured using MetaQUAST V5.0.2.[45] Alignment were then generated using Bowtie2 V2.2.3 [parameters: —very-sensitive-local] and reformatted them as bamfiles using samtools V1.0. Depth files were created using MetaBAT2’s built in “jgi_summarize_bam_contig_depths” function. Subsiquently, Contigs were binned into bacterial Metagenome-Assembled-Genomes on a sample-by-sample basis, using MetaBAT2 [parameters: –minContig 1500].[46]

After the bin identification step, genome bin quality was assessed using the CheckM V1.2.[47] workflow. High to medium-quality bins were de-replicated with deRep V3.2.2 [parameters: -p 15 -comp 50 -pa 0.9 -sa 0.95 -nc 0.30 -cm larger]. This produced a non-redundant bin database, which was then reformatted as an Xtree database [parameters: xtree BUILD k 29 comp 2]. Sample-by-

sample alignments and relative abundances were computed using the methods seen above. Bins were given taxonomic labels using GTDB-tK (v2.3.2).[48]

Viral contigs were identified using CheckV V0.8.1.[49] Viral abundance was quantified by first filtering for contigs that were labeled as either medium quality, high quality, or complete. The reduced contig database was then de-replicated using BLAST, and clustered at the 99% identity threshold as seen above, again using published approaches (https://github.com/snayfach/MGV/tree/master/ani_cluster).[40] Unique viral contigs were formatted as an Xtree database [parameters: xtree BUILD k 29 comp 0], and the sample-by-sample alignments and relative abundance quantities for each were computed with the same alignment and filtering methods seen above.

Annotation of viral contigs

Putative assembled viral genomes were annotated using a Hidden Markov Model (HMM) based approach (https://github.com/b-tierney/vironomy). The tool utilized for this purpose identifies single copy HMMs (from the pFam and TIGRFAM databases)[50,51] across a sizeable viral genome set, based on their overlap with the summation of the complete genomes of GenBank's viral database. The tool then attempts to assign taxonomy according to the count of overlapping genetic features. In this study, a sum total of 51 single-copy HMMs were used in the characterization of the assembled viral genomes' phylogenetic structure, and subsequently, the phylum closest matching the GenBank viruses based on their overlap were identified.

Gene catalog construction and functional annotation

Gene catalogs were generated using methods vetted in previous studies[52–54]. Bakta V1.5.1 was used to identify and mark putative Open-Reading-Frames (ORFs) across all assembled contigs.[55]

The non-redundant gene catalog was created by clustering predicted ORFs into homology-based sequence clusters using MMseqs2 V13.4511[56] [parameters: –easy-cluster –min-seq-id 0.3 -c 0.9]. Here, 30% was selected as an identity cutoff based on prior benchmarking of protein sequence annotations made by ourselves as well as other teams'.

The abundance of the representative sequences selected by MMseqs2 was computed by alignment of quality-controlled reads with Diamond V2.0.14.[57] From this, we determined the total number of hits and calculated gene relative abundance by dividing the number of reads aligned to a given gene by the count of aligned reads across all genes in a sample.

Metagenome-Association-Study

A mixed modeling approach was utilized in order to identify associations between spaceflight and microbial taxa. Associations for all the filtering cutoffs were calculated, data modalities (i.e., genes, viruses, bacteria), taxonomic levels (i.e., species vs genus, etc), algorithmic approaches (i.e., Kraken2, Xtree), and body sites (i.e., skin/nasal/oral) yielding a total of 248 different

individual association output files present in the resource. We fit a total of four model specifications:

1. $$ln(microbial\_feature\_abundance + minval) \sim Time * Armpit + Time * ToeWeb + Time * NapeofNeck + Time * Postauricular + Time * Forehead + Time * Bellybutton + Time * Glutealcrease + Time * Nasal + Time * T-zone + (1|Crew.ID)$$

2. $$ln(microbial\_feature\_abundance + minval) \sim Time * Skin + (1|Crew.ID)$$

3. $$ln(microbial\_feature\_abundance + minval) \sim Time * Oral + (1|Crew.ID)$$

4. $$ln(microbial\_feature\_abundance + minval) \sim Time * Nasal + (1|Crew.ID)$$

The key difference between the first and second modeling strategies is that the "skin" variable in the latter is encoded as a single, binary term indicating if a swab came from the skin or did not, whereas in the first each individual skin site is encoded as a separate variable. We report in Figure 6G-H output from the Kraken2 associations for model specifications 2-4.

Time is a variable encoded with three levels corresponding to the time of sampling relative to flight: pre-launch, mid-flight, and post-launch. The reference group was the mid-flight time point, indicating that any regression coefficients had to be interpreted relative to flight (i.e., a negative coefficient on the pre-launch time point implies that a feature was reduced prior to flight and increased in-flight). Each variable encoding a body site is binary encoding if a sample did or did not come from a particular region.

We used the lme4 package (v1.1) to compute associations between microbial features (i.e., taxa or gene) abundance and time as a function of spaceflight and bodysite. We estimated p-values on all models with the LmerTest (v3.1-3) packages using the default settings.[58,59] We adjusted for false positives by Benjaini-Hochberg adjustment and used a q-value cutoff point of 0.05 to gauge significance.

Cut&Run Analysis

Reads were preprocessed for quality control using FastQC[60] followed by trimmomatic adapter read trimming[61] and Bowtie2 alignment[62] to our STAR generated reference genome[63] and e.coli ASM484v2 genome assembly for spike-in reference. Paired ends were aligned end to end for mapping of inserts 10-700bp in length. The spike-in controls were aligned and a scaling factor was calculated for each sample. Duplicates were marked using DeDup and samtools was used to sort and index resulting BAM files. Alignments were filtered into two classes of reads, fragments less than 120bp and all fragment sizes. Paired-end bedgraphs were then generated first using bedtools bamtobed and then bedtools genomecov to convert bed files into bedgraphs and normalized to spike-in control using the scaling factor[64]. UCSC bedGraphToBigWig was used to generate bigwig tracks from the final alignments for visualization on IGV. DeepTools bamCoverage was also used to generate normalized bigwig files to reads per genome coverage at 1x depth using the effective genome size for comparison[65]. MACS2[66] was used to call peaks from normalized bedgraph files

using -f BAMPE, qvalue < 0.05, -B --SPRM, and --broad depending on what histone PTM was being analyzed. SEACR[67] was also used for peak calling with both relaxed and stringent settings and with and without normalization to control IgG input bedgraph. The called peaks were then annotated with genomic features using HOMER[68]. A MultiQC[17] report was generated for the individual samples to assess various quality metrics before merging replicate BAM files and performing the above steps to generate bedgraph, bigWig, and called peaks. Matrices around a reference point were created using normalized bigWig files and GencodeV36 TSS bed, hg38 Cage Promoter bed, or hg38 Fantom Enhancer bed files were used to plot using deepTools plotHeatmap[65].

References

1. Feng, W. *et al.* NULISA: a proteomic liquid biopsy platform with attomolar sensitivity and high multiplexing. *Nat. Commun.* **14**, 7238 (2023).
2. Blume, J. E. *et al.* Rapid, deep and precise profiling of the plasma proteome with multi-nanoparticle protein corona. *Nat. Commun.* **11**, 3662 (2020).
3. Ferdosi, S. *et al.* Engineered nanoparticles enable deep proteomics studies at scale by leveraging tunable nano-bio interactions. *Proc. Natl. Acad. Sci. U. S. A.* **119**, e2106053119 (2022).
4. Hoshino, A. *et al.* Extracellular Vesicle and Particle Biomarkers Define Multiple Human Cancers. *Cell* **182**, 1044–1061.e18 (2020).
5. Chen, Q. *et al.* Accelerated transsulfuration metabolically defines a discrete subclass of amyotrophic lateral sclerosis patients. *Neurobiol. Dis.* **144**, 105025 (2020).
6. Chen, Q. *et al.* Rewiring of Glutamine Metabolism Is a Bioenergetic Adaptation of Human Cells with Mitochondrial DNA Mutations. *Cell Metab.* **27**, 1007–1025.e5 (2018).
7. Chen, Q. *et al.* Measurement of Melanin Metabolism in Live Cells by [U-13C]-L-Tyrosine Fate Tracing Using Liquid Chromatography-Mass Spectrometry. *J. Invest. Dermatol.* **141**, 1810–1818.e6 (2021).

8. Cawthon, R. M. Telomere length measurement by a novel monochrome multiplex quantitative PCR method. *Nucleic Acids Res.* **37**, e21 (2009).
9. Freed, D., Aldana, R., Weber, J. A. & Edwards, J. S. The Sentieon Genomics Tools - A fast and accurate solution to variant calling from next-generation sequence data. *bioRxiv* 115717 (2017) doi:10.1101/115717.
10. Danecek, P. *et al.* Twelve years of SAMtools and BCFtools. *Gigascience* **10**, (2021).
11. McLaren, W. *et al.* The Ensembl Variant Effect Predictor. *Genome Biol.* **17**, 122 (2016).
12. Cingolani, P. *et al.* A program for annotating and predicting the effects of single nucleotide polymorphisms, SnpEff: SNPs in the genome of Drosophila melanogaster strain w1118; iso-2; iso-3. *Fly* **6**, 80–92 (2012).
13. Hao, Y. *et al.* Integrated analysis of multimodal single-cell data. *Cell* **184**, 3573–3587.e29 (2021).
14. Stuart, T. *et al.* Comprehensive Integration of Single-Cell Data. *Cell* **177**, 1888–1902.e21 (2019).
15. Gamaarachchi, H. *et al.* GPU accelerated adaptive banded event alignment for rapid comparative nanopore signal analysis. *BMC Bioinformatics* **21**, 343 (2020).
16. Leger, A. & Leonardi, T. pycoQC, interactive quality control for Oxford Nanopore Sequencing. *J. Open Source Softw.* **4**, 1236 (2019).
17. Ewels, P., Magnusson, M., Lundin, S. & Käller, M. MultiQC: summarize analysis results for multiple tools and samples in a single report. *Bioinformatics* **32**, 3047–3048 (2016).
18. Love, M. I., Soneson, C. & Patro, R. Swimming downstream: statistical analysis of differential transcript usage following Salmon quantification. *F1000Res.* **7**, 952 (2018).
19. Patro, R., Duggal, G., Love, M. I., Irizarry, R. A. & Kingsford, C. Salmon provides fast and

bias-aware quantification of transcript expression. *Nat. Methods* **14**, 417–419 (2017).

20. Robinson, M. D., McCarthy, D. J. & Smyth, G. K. edgeR: a Bioconductor package for differential expression analysis of digital gene expression data. *Bioinformatics* **26**, 139–140 (2010).
21. Li, H. Minimap2: pairwise alignment for nucleotide sequences. *Bioinformatics* **34**, 3094–3100 (2018).
22. Liao, Y., Smyth, G. K. & Shi, W. featureCounts: an efficient general purpose program for assigning sequence reads to genomic features. *Bioinformatics* **30**, 923–930 (2014).
23. Love, M. I., Huber, W. & Anders, S. Moderated estimation of fold change and dispersion for RNA-seq data with DESeq2. *Genome Biol.* **15**, 550 (2014).
24. Varet, H., Brillet-Guéguen, L., Coppée, J.-Y. & Dillies, M.-A. SARTools: A DESeq2- and EdgeR-Based R Pipeline for Comprehensive Differential Analysis of RNA-Seq Data. *PLoS One* **11**, e0157022 (2016).
25. Hendra, C. *et al.* Detection of m6A from direct RNA sequencing using a Multiple Instance Learning framework. *bioRxiv* 2021.09.20.461055 (2021) doi:10.1101/2021.09.20.461055.
26. Akalin, A. *et al.* methylKit: a comprehensive R package for the analysis of genome-wide DNA methylation profiles. *Genome Biol.* **13**, R87 (2012).
27. Välikangas, T., Suomi, T. & Elo, L. L. A systematic evaluation of normalization methods in quantitative label-free proteomics. *Brief. Bioinform.* **19**, 1–11 (2018).
28. Zhang, X. *et al.* Proteome-wide identification of ubiquitin interactions using UbIA-MS. *Nat. Protoc.* **13**, 530–550 (2018).
29. Ritchie, M. E. *et al.* limma powers differential expression analyses for RNA-sequencing and microarray studies. *Nucleic Acids Res.* **43**, e47 (2015).

30. Ritchie, M. E. *et al.* Empirical array quality weights in the analysis of microarray data. *BMC Bioinformatics* **7**, 261 (2006).

31. Perkins, D. N., Pappin, D. J. C. & Creasy, D. M. Probability-based protein identification by searching sequence databases using mass spectrometry data. *Electrophoresis* (1999).

32. Uhlén, M. *et al.* Proteomics. Tissue-based map of the human proteome. *Science* **347**, 1260419 (2015).

33. Korotkevich, G. *et al.* Fast gene set enrichment analysis. *bioRxiv* 060012 (2021) doi:10.1101/060012.

34. Hänzelmann, S., Castelo, R. & Guinney, J. GSVA: gene set variation analysis for microarray and RNA-seq data. *BMC Bioinformatics* **14**, 7 (2013).

35. Bushnell, B. BBTools software package. *URL http://sourceforge. net/projects/bbmap* **578**, 579 (2014).

36. Yost, S., Duran-Pinedo, A. E., Teles, R., Krishnan, K. & Frias-Lopez, J. Functional signatures of oral dysbiosis during periodontitis progression revealed by microbial metatranscriptome analysis. *Genome Med.* **7**, 27 (2015).

37. Hillmann, B. *et al.* Evaluating the Information Content of Shallow Shotgun Metagenomics. *mSystems* **3**, (2018).

38. Wood, D. E., Lu, J. & Langmead, B. Improved metagenomic analysis with Kraken 2. *Genome Biol.* **20**, 257 (2019).

39. Al-Ghalith, G. & Knights, D. BURST enables mathematically optimal short-read alignment for big data. *bioRxiv* 2020.09.08.287128 (2020) doi:10.1101/2020.09.08.287128.

40. Nayfach, S. *et al.* Metagenomic compendium of 189,680 DNA viruses from the human gut microbiome. *Nat Microbiol* **6**, 960–970 (2021).

41. Altschul, S. F., Gish, W., Miller, W., Myers, E. W. & Lipman, D. J. Basic local alignment search tool. *J. Mol. Biol.* **215**, 403–410 (1990).

42. Lu, J., Breitwieser, F. P., Thielen, P. & Salzberg, S. L. Bracken: estimating species abundance in metagenomics data. *PeerJ Comput. Sci.* **3**, e104 (2017).

43. Dixon, P. VEGAN, a package of R functions for community ecology. *J. Veg. Sci.* **14**, 927–930 (2003).

44. Nurk, S., Meleshko, D., Korobeynikov, A. & Pevzner, P. A. metaSPAdes: a new versatile metagenomic assembler. *Genome Res.* **27**, 824–834 (2017).

45. Mikheenko, A., Saveliev, V. & Gurevich, A. MetaQUAST: evaluation of metagenome assemblies. *Bioinformatics* **32**, 1088–1090 (2016).

46. Kang, D. D. *et al.* MetaBAT 2: an adaptive binning algorithm for robust and efficient genome reconstruction from metagenome assemblies. *PeerJ* **7**, e7359 (2019).

47. Parks, D. H., Imelfort, M., Skennerton, C. T., Hugenholtz, P. & Tyson, G. W. CheckM: assessing the quality of microbial genomes recovered from isolates, single cells, and metagenomes. *Genome Res.* **25**, 1043–1055 (2015).

48. Chaumeil, P.-A., Mussig, A. J., Hugenholtz, P. & Parks, D. H. GTDB-Tk: a toolkit to classify genomes with the Genome Taxonomy Database. *Bioinformatics* (2019) doi:10.1093/bioinformatics/btz848.

49. Nayfach, S. *et al.* CheckV assesses the quality and completeness of metagenome-assembled viral genomes. *Nat. Biotechnol.* **39**, 578–585 (2021).

50. Finn, R. D. *et al.* Pfam: the protein families database. *Nucleic Acids Res.* **42**, D222–30 (2014).

51. Haft, D. H., Selengut, J. D. & White, O. The TIGRFAMs database of protein families.

Nucleic Acids Res. **31**, 371–373 (2003).

52. Zimmerman, S., Tierney, B. T., Patel, C. J. & Kostic, A. D. Quantifying shared and unique gene content across 17 microbial ecosystems. *bioRxiv* 2022.07.19.500741 (2022) doi:10.1101/2022.07.19.500741.
53. Coelho, L. P. *et al.* Towards the biogeography of prokaryotic genes. *Nature* (2021) doi:10.1038/s41586-021-04233-4.
54. Tierney, B. T. *et al.* The Landscape of Genetic Content in the Gut and Oral Human Microbiome. *Cell Host Microbe* **26**, 283–295.e8 (2019).
55. Schwengers, O. *et al.* Bakta: rapid and standardized annotation of bacterial genomes via alignment-free sequence identification. *Microb Genom* **7**, (2021).
56. Steinegger, M. & Söding, J. MMseqs2 enables sensitive protein sequence searching for the analysis of massive data sets. *Nat. Biotechnol.* **35**, 1026–1028 (2017).
57. Buchfink, B., Xie, C. & Huson, D. H. Fast and sensitive protein alignment using DIAMOND. *Nat. Methods* **12**, 59–60 (2015).
58. Bates, D., Mächler, M., Bolker, B. & Walker, S. Fitting Linear Mixed-Effects Models Using lme4. *J. Stat. Softw.* **67**, 1–48 (2015).
59. Kuznetsova, A., Brockhoff, P. B. & Christensen, R. H. B. lmerTest Package: Tests in Linear Mixed Effects Models. *J. Stat. Softw.* **82**, 1–26 (2017).
60. Andrews, S. & Others. FastQC: a quality control tool for high throughput sequence data. Preprint at (2010).
61. Bolger, A. M., Lohse, M. & Usadel, B. Trimmomatic: a flexible trimmer for Illumina sequence data. *Bioinformatics* **30**, 2114–2120 (2014).
62. Langmead, B. & Salzberg, S. L. Fast gapped-read alignment with Bowtie 2. *Nat. Methods* **9**,

357–359 (2012).

63. Dobin, A. *et al.* STAR: ultrafast universal RNA-seq aligner. *Bioinformatics* **29**, 15–21 (2013).

64. Quinlan, A. R. & Hall, I. M. BEDTools: a flexible suite of utilities for comparing genomic features. *Bioinformatics* **26**, 841–842 (2010).

65. Ramírez, F. *et al.* deepTools2: a next generation web server for deep-sequencing data analysis. *Nucleic Acids Res.* **44**, W160–5 (2016).

66. Zhang, Y. *et al.* Model-based analysis of ChIP-Seq (MACS). *Genome Biol.* **9**, R137 (2008).

67. Meers, M. P., Tenenbaum, D. & Henikoff, S. Peak calling by Sparse Enrichment Analysis for CUT&RUN chromatin profiling. *Epigenetics Chromatin* **12**, 42 (2019).

68. Heinz, S. *et al.* Simple combinations of lineage-determining transcription factors prime cis-regulatory elements required for macrophage and B cell identities. *Mol. Cell* **38**, 576–589 (2010).
