# Supplementary material for: The Space Omics and Medical Atlas (SOMA) and international astronaut biobank
Source: Nature. 2024 Jun 11;632(8027):1145–54. doi: 10.1038/s41586-024-07639-y (PMC11357981; doi:10.1038/s41586-024-07639-y)
Supplement: Supplementary file 2 — Reporting Summary [file 41586_2024_7639_MOESM2_ESM.pdf]

nature portfolio

Corresponding author(s): Eliah G. Overbey
Cem Meydan
Christopher E. Mason

Last updated by author(s): May 23, 2024

Reporting Summary

Nature Portfolio wishes to improve the reproducibility of the work that we publish. This form provides structure for consistency and transparency in reporting. For further information on Nature Portfolio policies, see our Editorial Policies and the Editorial Policy Checklist.

Statistics

For all statistical analyses, confirm that the following items are present in the figure legend, table legend, main text, or Methods section.

n/a	Confirmed	
☐	☒	The exact sample size (n) for each experimental group/condition, given as a discrete number and unit of measurement
☐	☒	A statement on whether measurements were taken from distinct samples or whether the same sample was measured repeatedly
☐	☒	The statistical test(s) used AND whether they are one- or two-sided<br>*Only common tests should be described solely by name; describe more complex techniques in the Methods section.*
☐	☒	A description of all covariates tested
☐	☒	A description of any assumptions or corrections, such as tests of normality and adjustment for multiple comparisons
☐	☒	A full description of the statistical parameters including central tendency (e.g. means) or other basic estimates (e.g. regression coefficient) AND variation (e.g. standard deviation) or associated estimates of uncertainty (e.g. confidence intervals)
☐	☒	For null hypothesis testing, the test statistic (e.g. F, t, r) with confidence intervals, effect sizes, degrees of freedom and P value noted<br>*Give P values as exact values whenever suitable.*
☒	☐	For Bayesian analysis, information on the choice of priors and Markov chain Monte Carlo settings
☒	☐	For hierarchical and complex designs, identification of the appropriate level for tests and full reporting of outcomes
☒	☐	Estimates of effect sizes (e.g. Cohen's d, Pearson's r), indicating how they were calculated

Our web collection on statistics for biologists contains articles on many of the points above.

Software and code

Policy information about availability of computer code

Data collection	No software was used for data collection
Data analysis	ani-cluster (https://github.com/snayfach/MGV/tree/master/ani_cluster)<br>Bakta (v1.5.1)<br>bbtools (v38.92)<br>BCFtools (v1.9)<br>bedtools (v2.29.2)<br>Bowtie2 (v2.2.3)<br>bracken (v2.6.2)<br>BWA MEM (v0.7.15)<br>Cell Ranger (v6.1.1)<br>CheckV (v0.8.1)<br>CheckM (v1.2)<br>deepTools (v3.5.1)<br>DESeq2 (v1.36.0)<br>Diamond (v2.0.14)<br>FastQC (v0.11.9)<br>fgsea (v1.22)<br>GSVA (v1.42.0)<br>HOMER (v4.11)<br>Kraken2 (v2.1.2)

limma (v3.52)
m6Anet (v1.1.1)
MACS2 (v2.2.6)
MassProfinder 8.0
MetaQUAST (v5.0.2)
MetaSPAdes (v3.14.3)
methylKit (v3.18)
minimap2 (v2.24)
MMseqs2 (v13.4511)
MultiQC (v1.13)
pheatmap (v1.0)
PycoQC (v2.5.0.21)
samtools (v1.0, v1.9)
SARTools (v1.8.1)
SEACR (v1.3)
Sentieon TNscope RNA-seq variant pipeline (v202010)
Seurat (v4.2.0)
SnpEff (v4.3)
subread (v2.0.1)
trimmomatic (v0.39)
Varient-Effect-Predictor VEP (v107)
vegan (v2.6.2)
VGenes (vPre-release3)
vironomy (https://github.com/b-tierney/vironomy)
XTree (v0.92i)

Additional codes and scripts can be found in https://github.com/eliah-o/inspiration4-omics

For manuscripts utilizing custom algorithms or software that are central to the research but not yet described in published literature, software must be made available to editors and reviewers. We strongly encourage code deposition in a community repository (e.g. GitHub). See the Nature Portfolio guidelines for submitting code & software for further information.

Data

Policy information about availability of data

All manuscripts must include a data availability statement. This statement should provide the following information, where applicable:

- Accession codes, unique identifiers, or web links for publicly available datasets
- A description of any restrictions on data availability
- For clinical datasets or third party data, please ensure that the statement adheres to our policy

Datasets have been uploaded to the NASA Open Science Data Repository (osdr.nasa.gov; comprised of NASA GeneLab and the NASA Ames Life Sciences Data Archive)

OSD-569
https://osdr.nasa.gov/bio/repo/data/studies/OSD-569/
OSD-570
https://osdr.nasa.gov/bio/repo/data/studies/OSD-570/
OSD-571
https://osdr.nasa.gov/bio/repo/data/studies/OSD-571/
OSD-572
https://osdr.nasa.gov/bio/repo/data/studies/OSD-572/
OSD-573
https://osdr.nasa.gov/bio/repo/data/studies/OSD-573/
OSD-574
https://osdr.nasa.gov/bio/repo/data/studies/OSD-574/
OSD-575
https://osdr.nasa.gov/bio/repo/data/studies/OSD-575/
OSD-630
https://osdr.nasa.gov/bio/repo/data/studies/OSD-630/
OSD-656
https://osdr.nasa.gov/bio/repo/data/studies/OSD-656/

In addition the JAXA Cell-Free Epigenome (CFE) Study data and results were obtained from accession OSD-530.
https://osdr.nasa.gov/bio/repo/data/studies/OSD-530/

Processed data are also available for visualization in the following web portals.
https://soma.weill.cornell.edu/apps/SOMA_Browser/
https://soma.weill.cornell.edu/apps/I4_Multiome/
https://soma.weill.cornell.edu/apps/I4_Microbiome/

Human research participants

Policy information about studies involving human research participants and Sex and Gender in Research.

Reporting on sex and gender	Sex information was collected and annotated in the NASA OSDR repository for each sample collected.
Population characteristics	The crew member composition was of two races and ages ranged from 29-51.
Recruitment	Participants were recruited by SpaceX and mission commander Jared Isaacman through various task-specific criteria and raffle processes. There are no self-selection biases relevant to sample the collection protocols outlined in this paper.
Ethics oversight	All subjects were consented at an informed consent briefing (ICB) at SpaceX (Hawthorne, CA), and samples were collected and processed under the approval of the Institutional Review Board (IRB) at Weill Cornell Medicine, under Protocol 21-05023569. All crew members have consented for data and sample sharing.

Note that full information on the approval of the study protocol must also be provided in the manuscript.

Field-specific reporting

Please select the one below that is the best fit for your research. If you are not sure, read the appropriate sections before making your selection.

☒ Life sciences ☐ Behavioural & social sciences ☐ Ecological, evolutionary & environmental sciences

For a reference copy of the document with all sections, see nature.com/documents/nr-reporting-summary-flat.pdf

Life sciences study design

All studies must disclose on these points even when the disclosure is negative.

Sample size	No sample size calculations were performed. The entire Inspiration4 crew was profiled, which was limited by the size of the Dragon capsule (n=4).
Data exclusions	No data has been excluded.
Replication	Replication tests are difficult as mission parameters cannot be repeated. Where possible, data validation was performed via western blots to validate proteomic findings. Western blots were done only once due to limited material.
Randomization	No randomizations were performed, all of the subjects were profiled longitudinally and there were no experimental groups or interventions that separated the subjects.
Blinding	Blinding was not possible because all subjects were astronauts in the same crew.

Reporting for specific materials, systems and methods

We require information from authors about some types of materials, experimental systems and methods used in many studies. Here, indicate whether each material, system or method listed is relevant to your study. If you are not sure if a list item applies to your research, read the appropriate section before selecting a response.

Materials & experimental systems

n/a	Involved in the study	
☐	☒	Antibodies
☒	☐	Eukaryotic cell lines
☒	☐	Palaeontology and archaeology
☒	☐	Animals and other organisms
☒	☐	Clinical data
☒	☐	Dual use research of concern

Methods

n/a	Involved in the study	
☒	☐	ChIP-seq
☒	☐	Flow cytometry
☒	☐	MRI-based neuroimaging

Antibodies

Antibodies used	Abcam H3K27ac (CAT:ab245911)<br>Epicypher H3K4me1 (SKU:13-0040)

Cutana Rabbit IgG antibody (SKU: 13-0042)

Cell Signaling, human CD9 (rabbit monoclonal IgG, CAT:13174S)
Santa Cruz Biotechnology, Galectin 3 Binding Protein (LGALS3BP) (mouse monoclonal IgG, CAT:sc-374541)
Abcam, Fibronectin (FN1) (rabbit polyclonal IgG, CAT:ab2413)
Cell Signaling, β-actin (rabbit monoclonal IgG, CAT:8457S)
Jackson Laboratory, horseradish peroxidase (HRP)-labeled IgG goat anti-rabbit or goat anti-mouse

Validation

H3K27ac and H3K4me1 antibodies were compared to the Cutana Rabbit IgG antibody, which served as a negative control.

Information from the manufacturer:
Abcam H3K27ac (CAT:ab245911)
This antibody meets the Abcam promise guarantee for immunocytochemistry and immunofluorescence applications. and manufactrer notes it specifically binds to K27ac alone and also when S28 is phosphorylated.

Epicypher H3K4me1 (SKU:13-0040)
This antibody meets EpiCypher's "SNAP-ChIP® Certified" criteria for specificity and efficient target enrichment in a ChIP experiment (<20% cross-reactivity across the panel, >5% recovery of target input). This antibody binds to H3K4me1 and no significant cross reactivity with other lysine methylations in the EpiCypher SNAP-ChIP K-MetStat Panel (EpiCypher 19-1001) is detected.

Cell Signaling human CD9 antibody is noted as recognizing endogenous levels of total human CD9 protein and certified for western blot use by the manufacturer.

Santa Cruz Biotechnology Galectin 3 Binding Protein antibody is noted as being suitable for detection of Galecting-3BP/Mac-2BP of mouse, rat and human origin by western blot use by the manufacturer.

Abcam Fibronectin antibody is covered by the Abcam guarantee for use in Western blot and the manufacturer notes that it detects a band of approximately 285 kDa (predicted molecular weight: 262 kDa).

Cell Signaling β-actin antibody is marked for Western blot use, and the manufacturer notes that iut recognizes endogenous levels of total β-actin protein. Due to the high sequence identity between the cytoplasmic actin isoforms, β-actin and cytoplasmic γ-actin, this antibody may cross-react with cytoplasmic γ-actin. It does not cross-react with α-skeletal, α-cardiac, α-vascular smooth, or γ-enteric smooth muscle isoforms. Species Reactivity: Human, Mouse, Rat, Monkey, D. melanogaster, Zebrafish
